# Does Propylthiouracil Increase the Gentamicin-Induced Nephrotoxicity In Rat?

**Published:** 2013-11

**Authors:** Gholamreza Sepehri, Amin Derakhshanfar, Leila Saburi

**Affiliations:** 1 Neuroscience Research Center, Kerman University of Medical Sciences, Kerman, Iran; 2 Department of Pathology, Faculty of Veterinary Medicine, Shahid Bahonar University of Kerman, Kerman, Iran; 3 ^3^aculty of Veterinary Medicine, Shahid Bahonar University of Kerman, Kerman, Iran

**Keywords:** Gentamicin, Nephrotoxicity, Propylthiouracil, Rat

## Abstract

***Objective(s):*** The aim of this study was to evaluate the effect of subacute administration of propylthiouracil (PTU) on gentamicin (GM)-induced nephrotoxicity in male rats.

***Materials and Methods:*** Male Wistar rats were divided into 4 experimental groups as follow: (1) Control group: isotonic saline (1 ml/kg, IP. for 18 d), (2) GM group: 100 mg/kg, IP for 8 d, (3) PTU group: PTU (10 mg/kg, IP for 18 d.) and (4) PTU + GM group: GM (100 mg/kg, IP. for 8d) and PTU (10 mg/kg, IP. for 18 d). Blood sample was taken from all animals and then the animals were sacrificed under light ether anesthesia on the day after the last injection. Sera were separated and were used to measure the urea and creatinine. Microscopic evaluation of renal injury was performed using a semiquantitative scale to evaluate the degree of tubular necrosis.

***Results: ***GM markedly increased serum urea and creatinine, as well as acute tubular necrosis (ATN), glomerular atrophy, hyaline casts formation in tubular lumen, interstitial nephritis and infiltration of inflammatory cells. PTU administration alone caused hyperemia and interstitial nephritis and infiltration of lymphocytic inflammatory cells in cortex but it had no marked effect on glomerular and tubular morphology and function. Co-administration of PTU and GM potentiates the GM-induced nephrotoxicity characterized by diffuse ATN; diffuse hyaline cast formation in lumen and infiltration of inflammatory cell in kidney tissues.

***Conclusion:*** Our data indicate that PTU potentiates GM-induced nephrotoxicity. The underlying mechanism(s) via which PTU potentiates GM renal toxicity remains to be elucidated.

## Introduction

Aminoglycoside antibiotics are the most commonly used antibiotics worldwide in the treatment of Gram-negative bacterial infections. However, aminoglycosides induce nephrotoxicity in 10-20% of therapeutic courses. Aminoglycoside-induced nephrotoxicity is characterized by slow rises in serum creatinine, tubular necrosis and marked decreases in glomerular filtration rate and ultra-filtration coefficient (1-2).

The widespread therapeutic use of the aminoglycoside antibiotic gentamicin (GM) is limited by its nephrotoxic side effect and oxidative damage, which can lead to acute renal failure (3-4).

Aminoglycosides are taken up in the epithelial cells of the renal proximal tubules and stay there for a long time, resulting in nephrotoxicity. Acidic phospholipids are considered as a binding site for aminoglycosides in the brush-border membrane of the proximal tubular cells (5). Receptor-mediated endocytosis plays an important role in accumulation of aminoglycosides in renal proximal tubule (6). GM increases the generation of reactive oxygen metabolites in renal cortical mitochondria which causes GM-induced acute renal failure in rats (7). Previous studies showed morphological and structural alterations of glomeruli and glomerular basement membrane as well as alterations of proximal tubules in adult rats exposed to high doses of GM (3). Multivariate analysis showed that increase in aminoglycoside therapy duration is the most important factor associated with development of toxicity (8). Also, multidrug therapy, renal failure, some drugs (antithyroid drugs, lithium, amiodarone , rifampicin) and the age of patients (≥70 years ) are other risk factors related to aminoglycoside nephrotoxicity (8-10).

It is of remarkable interest to find agents to reduce or protect from aminoglycoside nephrotoxicity effects. Some studies demonstrated that antioxidant agents including beta blockers, superoxide dismutase mimetic agents, some hormones, iron chelators, some vitamins and medicinal plants act as ameliorating agents (11-14) . 

Since GM-induced nephrotoxicity leads to increased urinary losses of carnitine, so carnitine deficiency is a risk factor and should be regarded as a considerable cause during the development of GM-induced acute renal failure (ARF) and is reversed by carnitine supplementation (15). 

Hyperthyroidism and thyrotoxicosis are among diseases which needs long duration of continuous therapy of thioamide antithyroid drugs such as propylthiouracil (PTU). Adverse reactions to PTU are uncommon, occurring in 1–5% of patients (16, 17). Mild leukopenia, fever, rash, and arthralgia are the common side-effects. However, more serious reactions including agranulocytosis, hepatitis, vasculitis, and a lupus-like syndrome, acute interstitial nephritis with acute renal failure are also reported (16-22). Patients using thioamide antithyroid drugs such as PTU may use aminoglycoside for a long duration, so the combination therapy may potentiate adverse effects of the two drugs, including renal toxicity. Since there is no report regarding the effect of co-administration of PTU and GM on renal function, the aim of this study was to evaluate the effect of subacute administration of PTU and GM on renal histopathology and biochemical parameters of male rats.

## Materials and Methods

***Animals***

Male Wistar rats were purchased from Neuroscience Research Center (Kerman, Iran). GM and PTU were purchased from Darupakhsh, Co. (Tehran, Iran).

Four Wistar rats, 250-300 g, in each cage were kept in a temperature-controlled room at 25 ± 1°C with 12:12-hour light-dark cycle with lights on at 07:00 am. The experiments were carried out during the light phase of the cycle. The animals had free access to commercial food for rodents (Teklad Rodent Diet, Iran) and drinking water. One week prior to any treatment, animals were housed and acclimatized in the controlled room. The animals received human care in compliance. All procedures were in accordance with guidelines for caring and using of laboratory animals in Neuroscience Research Center of Kerman University of Medical Sciences and the European Communities Council Directive of 24 November 1986 (86/609/EEC).

Rats were randomly divided into 4 experimental groups, each comprising 7 animals:

1. Control group: Rats received a daily IP injection of isotonic saline (0.1 ml/kg/day) for 18 days.

2. GM group: GM (100 mg/kg) was injected (IP) daily to rats for 8 days (23-25).

3. PTU group: PTU (10 mg/kg) was injected (IP) daily to rats for 18 consecutive days.

4. PTU + GM group: Rats received GM (100 mg/kg, IP) for 8 days and PTU (10 mg/kg, IP) for 18 days. 

***Biochemical measurements***

Blood sample was taken from all animals and then the animals were sacrificed under light ether anesthesia on the next day after the last injection. Sera were separated and were used to measure urea and creatinine using a commercial kit (Zist Chimi Co, Iran) based on manufacturer recommendation. Also, blood indices such as red blood count (RBC) and white blood count (WBC) were determined.

*Histopathological evaluation*

The kidneys were removed from the rats at the end of the experimental period and were cut in a sagittal section into two halves. Renal tissue was fixed in 10% buffered-formalin solution and embedded in paraffin. Paraffin kidney sections (5 mm) were prepared and stained with haematoxylin and eosin(H&E) (26). Using light microscope (Nikon, Tokyo, Japan), microscopic evaluation of renal injury was performed by two pathologists for whom animal grouping was not known. Renal sections were scored with a semiquantitative scale designed to evaluate the degree of tubular necrosis. Injury was graded on a 5-point scale: 0: normal kidney; 1: minimal damage (<5% involvement of the cortex or outer medulla); 2: mild damage (5–25% involvement of the cortex or outer medulla); 3: moderate damage (25–75% involvement of the cortex or outer medulla); 4: severe damage (>75% involvement of the cortex or outer medulla (26).

***Statistical analysis***

Data are expressed as mean ± SEM of at least 7 rats. Comparisons were performed between control and drug treated groups by student paired t-test and among different groups by one-way ANOVA followed by *post hoc* Tukey’s test. Histopathological scores were assigned as normal (0), mild (1), moderate (2), severe (3) and very severe (4). *P*-value < 0.05 was considered as statistically significant.

## Results

***Effect of GM administration on rat renal histopathology***

**Figure 1:**
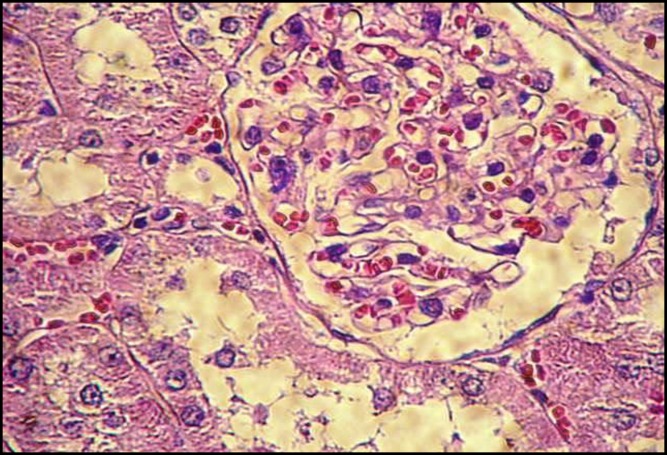
Normal morphological view of renal section in control group (H&E, ×400

**Figure 2:**
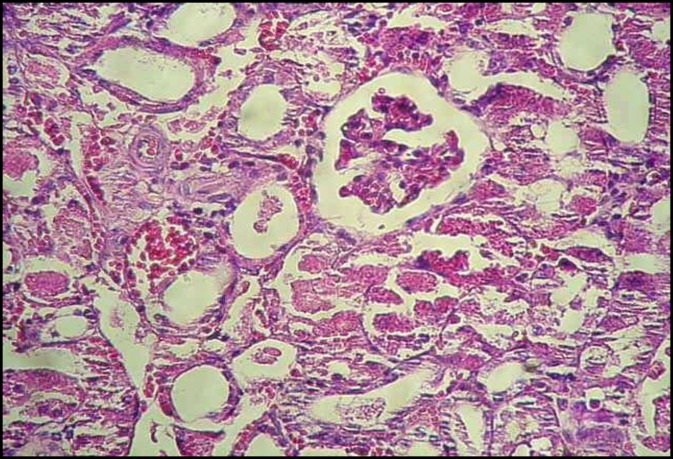
Glomerular atrophy and acute tubular necrosis of renal section in GM group (H&E, ×200

**Figure 3:**
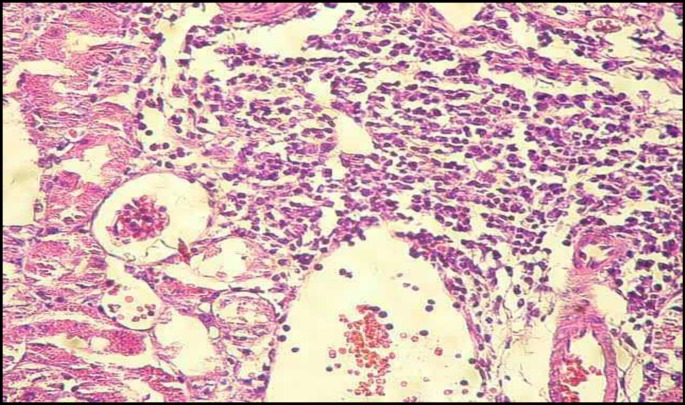
Lymphocytic interstitial nephritis of renal section in GM group (H&E, ×200

**Figure 4:**
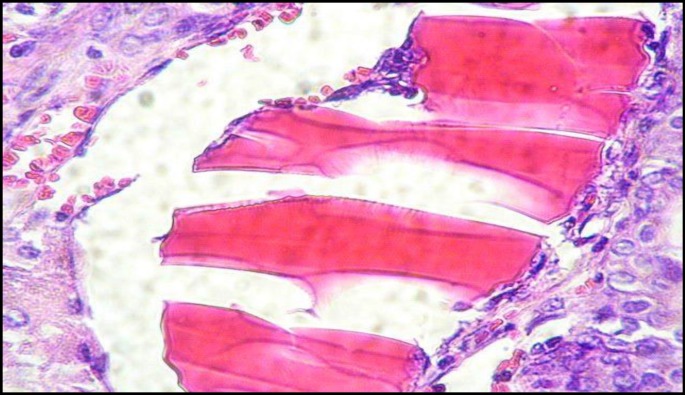
Hyaline cast in renal tubular lumen in GM group (H&E, ×400

Kidney tissues of the control group showed normal renal glomeruli surrounded by capsule, and normal convoluted tubules (Figure 1), however, histopathological scores of GM and PTU treated rats showed marked changes as compared to control group. GM treatment caused severe renal injury characterized by glomerular atrophy, acute tubular necrosis (ATN)(Figure 2), infiltration of lymphocytic inflammatory cells in cortex (Figure 3) hyaline cast formation in tubular lumen in some area (Figure 4), interstitial nephritis (Figure 3). Most of pathological scores in GM group were more severe and showed marked changes as compared to control rats (Table 1).

**Table 1:** Degrees of histopathological injuries of kidney sections in rats treated with GM, PTU and GM co-administration with PTU (n=28)

Parameters	Control	GM	PTU	GM+PTU
ATN	0	3	0	4
Glomerular atrophy	0	3	0	2
Hyaline casts in tubular lumen	0	2	0	4
Interstitial nephritis	0	2	2	0
Hyperemia	0	2	1	0
Lipofuscin pigments	0	0	2	0
Infiltration of inflammatory cells	0	2	1	2
Interstitial fibrosis	0	0	1	1

PTU (10 mg/kg for 18 d) was injected intraperitoneally (IP) alone or in combination with GM (80 mg/kg, IP for 8 d). Control rats received saline. GM= gentamicin, PTU= propylthiouracil, ATN= acute tubular necrosis 0 = normal, 1= mild, 2=moderate, 3= severe, 4 = very severe

***Effect of PTU administration on rat renal histopathology***

**Figure 5:**
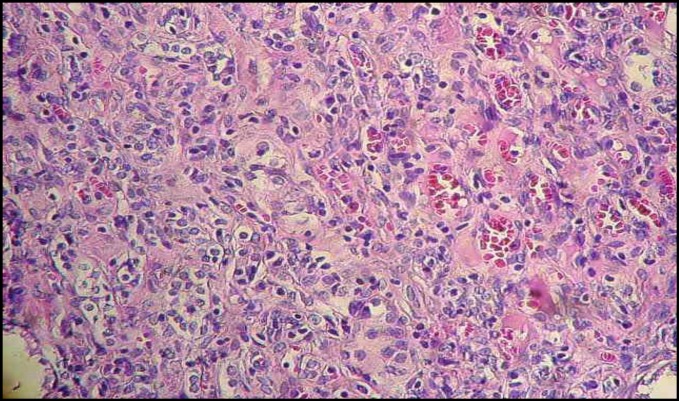
Hyperemia and interstitial fibrosis in renal section of PTU group (H&E, ×200

PTU administration (10 mg/kg, IP for 18 d) caused moderate interstitial nephritis (Figure 3), interstitial fibrosis and hyperemia, (Figure 5), infiltration of lymphocytic inflammatory cells in cortex (Figure 3), (Table 1).

***Effect of PTU administration with GM on rat renal histopathology***

The administration of PTU with GM caused diffuse ATN, glomelular atrophy, interstitial fibrosis, diffuse hyaline cast formation in tubular lumen and infiltration of lymphocytic inflammatory cells in cortex (Figures 2-5). Most of pathological scores in PTU+ GM treated rats were more severe and showed marked changes as compared to GM and control rats (Table 1).

***Effect of GM and PTU administration on biochemical parameters in serum***

GM treatment for eight days resulted in significant increase in serum creatinine and blood urea nitrogen compared to control rats (*P*<0.0001) but it had no significant effect on RBC, WBC, hemoglobin, neutrophils and monocytes. PTU administration had no significant effect on urea and creatinine level, but PTU administration alone /or with GM caused a significant increase in WBC and lymphocytes as compared to control (*P*<0.0001). The co-administration of GM with PTU resulted in a significant increase in both urea and creatinine compared to control and GM treated rats (*P*<0.0001and *P*<0.005, respectively) (Table 2).

## Discussion

The aim of the present study was to determine the effects of co-administration of GM with PTU on renal histopathology of rats. The results indicated that GM administration caused a significant increase in serum urea and creatinine level, as well as histopathological changes (ATN, glomerular atrophy; hyaline casts in tubular lumen, interstitial nephritis, infiltration of inflammatory cells) in renal tissues. PTU administration also caused interstitial nephritis and hyperemia.

The combination therapy of GM and PTU caused a marked increase in serum urea and creatinine as compared to GM treated rats. Also, combination therapy of GM and PTU caused massive renal injury characterized by diffuse ATN, diffuse hyaline cast in lumen, glomerular atrophy, infiltration of lymphocytic inflammatory cells in the kidney. Also, GM administration alone/or in combination with PTU significantly increased the serum levels of urea and creatinine as compared to control.

Morphological and structural alterations of glomeruli and glomerular basement membrane as well as alterations of renal tubules characterized by glomerular atrophy, ATN, interstitial nephritis, hyaline casts in tubular lumen and hyperemia, increase in serum urea and creatinine in adult rats exposed to GM are similar to the previously reported results (2, 9, 23, 27, 28).

There are several mechanism(s) involved in GM toxicity. Some investigators suggested that superoxide anions play an important role in GM-mediated nephropathy and support the possible clinical use of low molecular weight synthetic superoxide dismutase mimetics in those conditions associated with over production of superoxide (11, 29-32). GM decreased the activities of catalase (CAT), glutathione peroxidase (GSHPx) and the level of glutathione (GSH), and increases both plasma malondialdehyde (MDA) and kidney MDA, as well as lipid hydroperoxide (LOOH) formation(33) , increase in matrix metalloprotease (MMP)-2, increase in kidney myeloperoxidase activity and lipid peroxidation (30, 34) and many antioxidants such as vitamins ( C , E and B6), selenium and many medicinal plants (e.g. garlic, Spirulina platensis, curcumin) and sesame oil may attenuates GM-induced renal oxidative damage in rats (12, 31, 33-40).

Although PTU administration alone caused hyperemia, interstitial nephritis and infiltration of lymphocytic inflammatory cells in cortex but it had no significant effect on glomerular and tubular morphology and function. However, the co-administration of PTU with GM potentiates the nephrotoxicity of GM in treated rats. The mechanism(s) by which PTU potentiates GM renal toxicity is not determined, however, since PTU administration caused interstitial nephritis, interstitial fibrosis and infiltration of inflammatory cell in kidney tissues, so the combination therapy of GM and PTU may show synergistic effect on renal injury which was characterized by diffuse ATN, diffuse hyaline cast in lumen and infiltration of inflammatory cell in kidney tissues. Our results are in agreement with the previous reports on the PTU-induced acute interstitial nephritis with acute renal failure requiring haemodialysis (19), anti-neutrophil cytoplasmic antibodies (ANCA), positive glomerulonephritis and IgA nephropathy in patient on PTU therapy (41). It was reported that chronic therapy with PTU has been associated with drug-induced tubulointerstitial nephritis with renal failure (17). Also, PTU therapy may cause diffuse proliferative lupus nephritis via altering immunological responses (42).

**Table 2:** Effect of PTU on GM-induced changes in serum creatinine, urea level and blood parameters in rats

Parameter	Control (n=7)	GM (n=7)	PTU (n=7)	GM +PTU (n=7)
Urea (mg/dl)	33.8±55	112*±18	45.2±7	132**±19
Creatinine (mg/dl)	0.63±0.1	1.47*±0.3	0.75±0.25	1.76**±0.2
Hemoglobin(g/dl)	12.9±2.8	11.5±0.5	12.55±0.77	12.11±0.45
RBC (10^6^/µl)	7.43±0.82	6.8±0.3	6.8±0.42	6.96±0.24
WBC(10^3^/µl)	8.93±0.88	7.45±0.72	14.65*±2.22	13.95*±0.96
Neutrophil(10^3^/µl)	2.2±0.26	1.9±0.4	3.73±0.58	2.28±0.3
Lymphocyte(10^3^/µl)	6.5±0.54	5.9±0.65	10.13*±1.7	9.83*±0.9

PTU (10 mg/kg for 18d) was injected (IP) alone or in combination with GM (80 mg/kg, IP for 8d). Control rats received saline. Results are Mean ± SEM of seven different samples.

C: Control, PTU: Propylthouracil**, **GM: gentamicin, RBC: red blood cell, WBC= white blood cell.* P<0.0001, significantly different from control; **P<0.005, significantly different from GM

Contrary to our result, Abraham *et al* (2005) reported that PTU attenuates acetaminophen-induced renal damage in rat (43). The mechanism of protection by PTU is probably not due to the sparing effect of non-protein thiol (approximately 95% of which is reduced glutathione), as similar depletion of renal glutathione was observed regardless of PTU pretreatment; other mechanisms are suggested (43). Also, PTU administration alone/or with GM caused a significant increase in WBC and lymphocytes as compared to control (*P*<0.0001). The underlying mechanism(s) is not known, however, it could be due to chronic inflammatory response which was characterized by diffuse infiltration of lymphocytic inflammatory cells in kidney tissues following PTU administration.

## Conclusion

In summary, this study showed that GM causes marked increase of serum urea and creatinine and moderate histological injury of renal in rat. PTU has synergistic effects with GM in inducing renal dysfunction in rat. 
